# Nutritional Status Impact on Hip Fracture Patients in a Rural Environment

**DOI:** 10.3390/nu16213622

**Published:** 2024-10-25

**Authors:** Ana Martín-Nieto, Pedro Chana-Valero, Jaime Ruiz-Tovar, Gema Escobar-Aguilar, María Simarro-González, Pablo Rodríguez-Bernal, Elena García-García

**Affiliations:** 1 San Juan de Dios Foundation, 28036 Madrid, Spain; amartinn@comillas.edu (A.M.-N.); pchana@comillas.edu (P.C.-V.); gemaescobar@comillas.edu (G.E.-A.); msimarro@comillas.edu (M.S.-G.); elenagg@comillas.edu (E.G.-G.); 2 Health Sciences Department, San Juan de Dios School of Nursing and Physical Therapy, Comillas Pontifical University, 28036 Madrid, Spain; 3 Dietetics and Nutrition Department, Hospital San Juan de Dios, 24010 León, Spain; pablo.rodriguez@sjd.es

**Keywords:** hip fracture, malnutrition, sarcopenia, vitamin D, albumin, blood ureic nitrogen, mortality predictors

## Abstract

(1) Background: Hip fractures are highly prevalent traumatic events with significant functional consequences, particularly among the older population. These fractures are associated with increased mortality, postoperative complications, and functional dependence. Medical and nutritional factors such as malnutrition and sarcopenia are crucial for predicting functional outcomes and mortality in these patients. This study aimed to assess the nutritional status, vitamin D levels, and sarcopenia, as well as their relationship with mortality, mean hospital stay, and 30-day-readmission rate, in patients with hip fracture who underwent surgery in a rural hospital setting. (2) Methods: A longitudinal retrospective study involving 124 patients who underwent hip fracture surgery in 2021 was conducted. Sociodemographic, surgical, and nutritional data, including vitamin D, albumin, and blood urea nitrogen (BUN) levels, were collected. (3) Results: The average age of the sample was 89.1 years, with a postoperative 30-day-mortality rate of 8.1% and an average hospital stay of 10.4 days. Vitamin D deficiency was present in 79.7% of patients, and a high prevalence of malnutrition was indicated by low albumin and elevated BUN levels. Elevated BUN levels and low vitamin D levels were associated with higher mortality. (4) Conclusions: Adequate nutritional assessment in patients with hip fracture is vital for identifying the risks of complications and mortality. Understanding the current nutritional status and its associated complications will aid in developing strategies to improve health and reduce complications in the future.

## 1. Introduction

In developed countries, hip fractures are one of the most prevalent traumatic events, with the most significant functional consequences for patients. According to the latest report from the Spanish National Hip Fracture Registry [1] in 2022, a total of 9553 cases were reported, with 97.8% requiring surgical intervention. Additionally, due to the increase in life expectancy, the number of patients is expected to increase in the coming years, with an estimated annual 500,000 acute hip fractures by 2040 [2,3,4].

Hip fractures are more prevalent in the older population, with an average age of 86.9 years [1]. The profile of a hip fracture patient according to the Spanish National Hip Fracture Registry is an older adult, often with cognitive impairment and frailty [1]. Furthermore, hip fractures are directly associated with increased mortality, prolonged hospital stays, post-surgical complications such as pressure ulcers, infections, cognitive decline, and especially dependency for daily activities and loss of mobility [5,6]. These complications, in turn, contribute to increased healthcare costs [7].

Medical factors play a critical role as predictors of poor functional outcomes and mortality in older patients with hip fracture. Cognitive impairment, the presence of multiple comorbidities, a high American Society of Anesthesiologists (ASA) grade, cognitive impairment, and poor pre-fracture functional status are highlighted as the most relevant factors. In relation to nutrition, malnutrition, high Body Mass Index (BMI), frailty, sarcopenia, serum albumin and folic acid level, osteoporotic treatment, and low hemoglobin levels are also crucial predictors [3].

Individuals who have experienced a hip fracture are at a higher risk of malnutrition or may become malnourished during their recovery, primarily due to the catabolic processes that occur in the body [8]. Additionally, they may present other geriatric nutritional problems, such as frailty and sarcopenia, which often overlap and reinforce each other [9]. This risk of malnutrition can be attributed to various factors, including social, psychological, physical, economic, medical, or cognitive aspects. The dietary intake of hospitalized individuals or those recovering from a hip fracture is often suboptimal, with high rates of energy–protein malnutrition [10]. The prevalence of malnutrition in patients with hip fracture is significant and shows considerable variability, depending on the measurement tools used [11].

Albumin is the most abundant protein in the human body. It is synthesized in the liver and is found in large proportions in the lymphocytes. The normal concentration in the human blood is between 3.5 and 5 g/dL, and it is essential for maintaining oncotic pressure and the proper distribution of body fluids between the intravascular and extravascular compartments [12]. Albumin is considered a nutritional marker with significant prognostic value. Various documents and clinical guidelines have indicated its role in protein malnutrition, and its severity has been graded based on its value [13,14]. Additionally, albumin has been used in nutritional screenings such as the hospital CIPA (Control of intake, Protein, and Anthropometry) and CONUT (Controlling Nutritional Status) [15,16].

Numerous epidemiological studies linked energy–protein malnutrition, combined with a deficiency of vitamins and minerals, to increased mortality [8], decreased functional independence [17], increased postoperative complications, prolonged hospitalization, readmission, loss or reduction in mobility [11,18], and even poorer wound healing or failure of internal fixation [19].

In addition to this potential malnutrition in older patients undergoing hip fracture surgery, vitamin D deficiency often results from reduced sun exposure, decreased synthesis of vitamin D3, and a reduction in renal 1-α-hydroxylase owing to declining renal function [20]. Some scientific studies showed that vitamin D deficiency is directly related to reduced muscle strength, bone loss, and a higher risk of falls and bone fractures [21,22]. Moreover, due to its relationship with bone metabolism, its deficiency can increase the risk of fractures, especially in the hip [23,24]. Sarcopenia, defined as a decrease in muscle mass, muscle strength, and neuromuscular function related to age, is associated with a higher risk of osteoporosis and falls, explaining why patients with hip fracture have higher prevalence rates than other hospitalized patients. Additionally, the coexistence of sarcopenia in patients can increase the risk of death and is considered an important prognostic factor for predicting mortality in hospitalized older patients [25].

Therefore, an adequate nutritional assessment of patients who have suffered a hip fracture is important. The following hypothesis was tested: elevated BUN levels and low vitamin D levels are associated with higher mortality, an increased mean length of stay, and an increased 30-day-readmission rate in hip fracture patients. This study aimed to assess the nutritional status, vitamin D levels, and sarcopenia, as well as their relationship with mortality, mean hospital stay, and 30-day-readmission rate, in older patients with hip fracture who underwent surgery in a rural hospital setting.

## 2. Materials and Methods

A longitudinal retrospective study was conducted, including all patients who underwent hip fracture surgery, a total of 124, during the year 2021 at a second-level Spanish hospital located in an area with wide geographic dispersion and social isolation.

Inclusion criteria were adults aged 65 and older, with a medical diagnosis of hip fracture (intracapsular, intertrochanteric, or subtrochanteric) confirmed by anteroposterior (AP) and lateral X-rays at the emergency department.

The sample size consisted of all patients who met the inclusion criteria during the year 2021.

### 2.1. Sociodemographic Variables

A significant portion of the variables collected in this study are part of the database adapted from the Spanish National Hip Fracture Registry. This multicentre registry collects information on the epidemiological, clinical, functional, and healthcare characteristics of patients with hip fracture in various hospitals across Spain. This registry allows for follow-up one month after hospital discharge, providing valuable data on the patients’ progress after intervention or hospital treatment [1].

Data regarding preoperative age, sex, fracture side, and fracture type were collected. Additionally, the mortality rate, mean hospital stay, and readmission rate were evaluated.

### 2.2. Surgical Procedure Variables

Regarding the surgical intervention, the following procedures were taken into account: intramedullary nail, hemiarthroplasty, and total prosthesis. Furthermore, the surgical risk variable was assessed through the ASA scale. This step is a classification consisting of 6 categories that aim to categorize patients based on their health or disease status [26].

### 2.3. Nutritional Value Variables

To evaluate the nutritional status of the patients, vitamin D, serum albumin, and blood urea nitrogen (BUN) levels were collected. These values were obtained from a peripheral blood puncture in the antecubital vein, collected after a fasting period of 8–12 h. The samples were drawn into a 6 mL Venojet^®^ II (Terumo; autosep^®^, Tokyo, Japan) tube. Biochemical determinations were performed at 37°, using a Roche/Hitachi 747 automatic analyzer and the corresponding reagents provided by Roche (Basel, Switzerland). All measurements were performed in the clinical analysis laboratory of the hospital. Vitamin D intake and calcium supplementation prior to the fracture were also included.

### 2.4. Ethical Aspects

This study was evaluated and approved by the Research Ethics Committee of Saint John of God Foundation with protocol code: P_2020_006. The database was anonymized in accordance with the criteria of the Organic Law 3/2018 (https://www.cliclaw.com/library/international-laws/spain/organic-law-32018-december-5-protection-personal-data-and-guarantee, accessed on 22 October 2024) on Data Protection and in compliance with the provisions of Regulation (EU) 2016/679 (https://eur-lex.europa.eu/legal-content/EN/TXT/?uri=CELEX%3A32016R0679, accessed on 22 October 2024), preventing the direct and indirect identification of participants and the processing of their data. Likewise, during this project, the guidelines and regulations of the Declaration of Helsinki were followed, especially those approved at the 64th General Assembly in Fortaleza (Brazil), as well as the national legislation in force regarding the analysis of persons, their protection, and confidentiality.

### 2.5. Statistical Analysis

All statistical analyses were performed using SPSS Version 28.0 (SPSS Inc., Chicago, IL, USA). Results are expressed as mean ± standard deviation in Gaussian variables and the median and interquartile range are expressed in non-Gaussian. Qualitative variables are defined by number and percentages. Student *t*-test and Mann–Whitney’s test were used to compare data between the groups.

Cut-off points were established using ROC curves, and sensibility and specificity were accordingly calculated. Nonparametric tests were used for non-Gaussian variables.

A *p* value < 0.05 was considered statistically significant.

## 3. Results

A total of 124 patients were included in this study, 92 females and 32 males, with a mean age of 89.1 ± 5.1 years. Hip fractures occurred on the left side in 59 (47.6%) patients and on the right side in 65 patients (52.4%). The fracture types are described in Table 1.

Due to previous analytical deficiencies of these micronutrients, 12 (9.7%) patients were taking calcium and 20 (16.1%) vitamin D orally.

### 3.1. Surgical Procedure

According to the advanced age of the patients, the anesthetic risk was high; 14 (11.3%) patients had an ASA surgical risk classification grade 2, 96 (77.4%) patients had grade 3, and 14 (11.3%) had grade 4. Consequently, 96.7% of the patients underwent surgery under regional anesthesia. The surgical procedures were intramedullary nail placement in 63 cases (50.8%), cementless partial prosthesis placement in 26 (20.9%), and cementless total prosthesis placement in 35 (28.2%) (Table 2). The mean delay to surgery was 82.02 ± 51.6 h (range 8–376 h) due to logistic aspects or for optimization of the patient.

30-day-mortality rate was 8.1% (10 patients). Mean hospital stay was 10.4 ± 4.0 days, and 30-day-readmission rate was 8%.

### 3.2. Nutritional Values

Mean preoperative albumin levels were 3.6 ± 0.4 g/dL, with 46 patients (37.1%) presenting hypoalbuminemia (Albumin < 3.4 g/dL).

The median vitamin D value was 8.4 ng/mL (Interquartile range 5.7–15.4 ng/mL), with 99 (79.7%) of the patients presenting deficiency (vitamin D < 20 ng/dL), 18 (14.6%) insufficiency (vitamin D 20–30 ng/dL) and only 7 (5.7%) with values within the normal range. Of the 20 patients taking vitamin D pre-fracture, only 5 (25%) showed normal serum vitamin D levels, 10 (50%) insufficient levels, and 5 (25%) deficient levels, demonstrating an inadequate supplementation.

Mean BUN (Blood Urea Nitrogen) levels were 30.5 ± 12.8 mg/dL, with 82.8% of fracture patients presenting elevated BUN values (BUN > 20 mg/dL), which are associated with sarcopenia.

### 3.3. Mortality Predictors

Postoperative mortality was associated with lower vitamin D levels (7.4 ± 2.7 ng/mL in the patients who died vs. 12.7 ± 9.6 ng/mL in the patients who survived; *p* = 0.006). A significant cut-off point could not be established.

**Figure 1:**
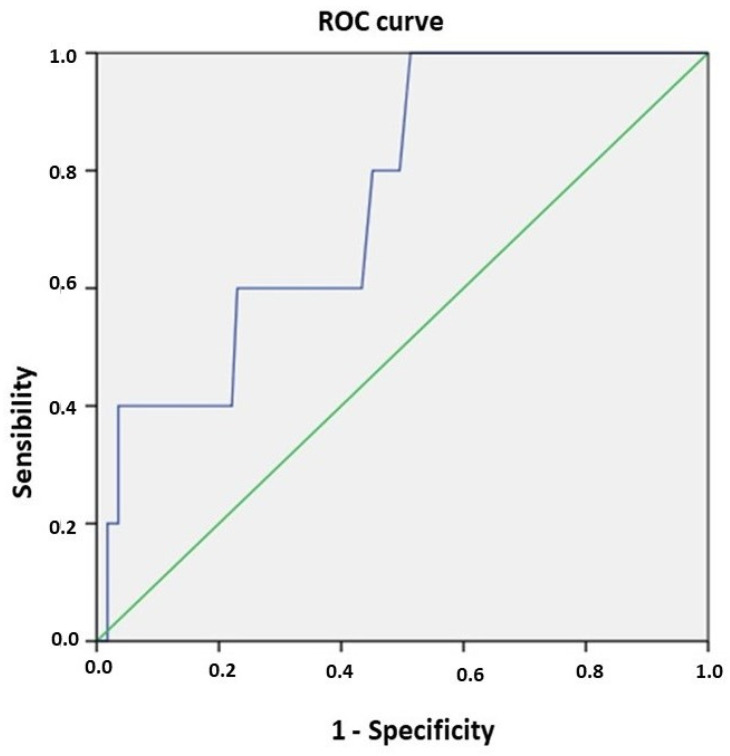
Sensitivity and specificity values of BUN for predicting mortality.

Postoperative mortality was also associated with higher BUN levels (44.1 ± 18.4 mg/dL in the patients who died vs. 30.4 ± 12.4 mg/dL in the patients who survived; *p* = 0.02). BUN values > 28.9 mg/dL had a sensitivity of 80% and a specificity of 54.9% for predicting postoperative mortality (AUC 0.755; CI95% (0.57–0.94); *p* = 0.05) (Figure 1).

## 4. Discussion

In terms of the sociodemographic results of the sample, it is worth noting that the mean age was close to 90 years. The average age recorded in our study was higher than that reported in similar studies. In a published review, an average age of 83 years was reported [27]. As the population ages, hip fractures are becoming more frequent and are associated with a high rate of morbidity and mortality. Additionally, the older the population, the greater the risk of protein malnutrition, which, in turn, increases the risk of complications and mortality [28].

Regarding the supplementation of the patients treated, a low percentage of them were taking calcium and vitamin D supplements before suffering the fracture. The vitamin D levels obtained were very low, and of the 20 patients taking vitamin D supplementation pre-fracture, only 25% had normal vitamin D levels, indicating that the supplementation was insufficient, or that the patients were not properly monitored. Various studies showed that prolonged supplementation with calcium and vitamin D appears to confer a substantial reduction in the risk of hip fracture in postmenopausal women [29].

Malnutrition in these patients was evaluated based on serum albumin levels. Serum albumin, in addition to being a nutritional marker, has been associated with inflammation. Various studies indicated that albumin is an inflammatory marker that can predict survival in patients with oncological pathology [30,31]. Numerous studies evaluated the nutritional status of patients with hip fracture through serum albumin values [30,31,32]. In the analyzed sample, low albumin values and hypoalbuminemia were found in 47% of patients. The low values obtained in this study are consistent with the reviewed scientific literature; studies showed that low values are directly related to an increase in complications and mortality [33,34]. Additionally, it was associated with higher rates of intubation, sepsis, and longer hospital stays compared to patients with albumin values within the normal range [35,36].

In 2020, a systematic review [11] was published that addressed the relationship between malnutrition and sarcopenia in older patients undergoing hip fracture surgery. The authors concluded that malnutrition and sarcopenia are very prevalent clinical aspects in these situations and have a negative impact on surgical outcomes. Therefore, clinicians are advised to understand and evaluate these aspects to improve patient recovery. In addition to understanding the nutritional problems of this population, it is recommended to carry out an intervention with a comprehensive approach that appropriately combines rehabilitation and nutritional interventions. Given the demonstrated importance of preoperative nutrition in this type of patient, it is recommended that it be properly evaluated in all patients and included as a variable to be measured in the National Hip Registry. Conducting appropriate screening in all patients will allow for adequate intervention to prevent negative consequences. Treatments aimed at improving the nutritional status of these patients focus primarily on the recovery from hip fracture by increasing the intake of energy, proteins, vitamins, or minerals, either independently or in combination [37]. Furthermore, such interventions can be administered orally, enterally, or intravenously [37]. Pedersen et al. [38] demonstrated that carbohydrate-enriched drinks and protein supplements administered 2 h before surgery could reduce catabolism and thus indirectly reduce possible postoperative complications. High-protein oral nutritional supplements have also proven to be effective, especially in the older population. These supplements were administered in most cases during the first week after the fracture up to the first month or even six months later [37]. Ekinci et al. [22] demonstrated that in older patients, a combined supplementation with vitamin D, calcium ß-hydroxy methylbutyrate (HMB), and proteins resulted in reduced immobility, accelerated wound healing, increased muscle strength, and reduced postoperative complications. According to the scientific literature, in patients over 65 years old, a vitamin D intake of 800–1000 IU/day is recommended to improve bone health and prevent fractures. Additionally, this vitamin D intake should be accompanied by a calcium intake of 1000 to 1200 mg/day. On the other hand, moderate sun exposure is recommended, avoiding peak intensity hours to reduce the risk of skin damage. It is also advisable to consume foods rich in vitamin D such as fatty fish, liver, egg yolk, and fortified dairy products [39].

The relationship between BUN and sarcopenia is multifaceted and varies across different populations. Higher BUN levels are generally associated with an increased risk of sarcopenia in community-dwelling older adults and patients with chronic conditions [40,41]. In the studied sample, 82.8% of the patients had high BUN levels, above 20 mg/dL, with an average of 30.5 mg/dL. These elevated values are consistent with the scientific literature on patients with hip fracture [42]. As mentioned in the Introduction, sarcopenia is associated with an increased risk of osteoporosis and falls, explaining why patients who have suffered a hip fracture have higher prevalence rates than other hospitalized patients [43]. In turn, the coexistence of sarcopenia in patients can increase mortality, being considered an important prognostic factor for predicting mortality in hospitalized older people [19]. In addition to an increase in mortality, the presence of sarcopenia is directly related to repeated falls, fractures, functional dependence, disability, increased hospital stay, the need for prolonged care, increased healthcare costs, and a decrease in quality of life [25].

In terms of mortality and hospital stay, the evaluated sample showed an 8.1% mortality rate and an average hospital stay of 10.4 days. The mortality rate in our sample was higher than what is indicated in the scientific literature, which places hospital mortality between 3% and 5% [44,45,46]. From our perspective, this high mortality rate is due to different factors. First, the advanced age of our sample was approximately 90 years. Advanced age is a known risk factor for increased mortality in hospitalized patients [47]. For every 10 years of increased age, the risk of hospital mortality almost doubles. However, as mentioned throughout the text, high rates of malnutrition and possible sarcopenia were found, which are directly related to the increase in hospital mortality in patients undergoing surgery for hip fracture. It should be noted that this study was based on a rural population, which makes the clinical follow-up of these patients difficult. This difficulty is evidenced by the fact that most patients who receive preoperative vitamin D supplementation do so at insufficient doses and therefore show analytical values, even in the range of insufficiency or deficiency. It is possible that these patients did not attend regular health checkups. This possibility would also apply to hypoalbuminemia and other nutritional deficiencies. Therefore, the rural population itself must be considered at an increased risk not only for hip fracture but also for perioperative complications and mortality. It should be noted that patients living in rural areas often have fewer medical facilities and fewer healthcare professionals, which can make it difficult to access adequate treatment and follow-up for hip fractures and vitamin D supplementation. Long distances and lack of transportation also make follow-ups difficult, resulting in a higher number of complications that cannot be adequately addressed. Additionally, people living in rural areas may have lower incomes and less access to educational resources about the importance of nutrition and vitamin D supplementation. This factor can also lead to a higher prevalence of nutritional deficiencies and postoperative complications, which may explain the results of this study [48,49].

Regarding the factors that predict postoperative mortality, the data agreed with those previously mentioned. High BUN levels are directly associated with increased mortality. Despite the fact that sarcopenia is very common in patients who have suffered a hip fracture, it is considered a preventable and treatable syndrome. Therefore, early diagnosis is important. In subsequent studies, given the importance of possible sarcopenia as a predictor of mortality, its inclusion in the assessment of all patients with hip fracture is recommended. In addition to measuring BUN, as in this study, other more specific diagnostic methods are recommended, such as grip strength, the SARC-F scale, dual-energy X-ray absorptiometry (DEXA), bioelectrical impedance analysis, or muscle ultrasound [25,50,51]. In addition to proper diagnosis, it is important to plan a treatment to improve outcomes. According to the literature, treatment for sarcopenia should include an appropriate combination of physical exercise and nutritional counselling [6]. Nutritional interventions that have shown benefits include the intake of branched-chain amino acids, vitamin D, whey protein, or supplementation with hydroxymethylbutyrate (HMB) [52,53].

The main strength of this study is the determination of a cut-off point of BUN > 28.9 mg/dL, which allows the determination of an increased risk of perioperative mortality in the immediate preoperative period. Clinicians must be aware of this risk and evaluate whether preoperative nutritional optimization is necessary before planning traumatic surgery.

A limitation of this study is that the malnutrition of the patients was evaluated using serum albumin, which can be affected by many other non-nutritional factors (disease, hospitalization, or therapeutic treatments). Therefore, in subsequent studies, the use of other tools to assess malnutrition, such as the Mini Nutritional Assessment-short form (MNA^®^ SF), is recommended. In addition, we only have three parameters to assess nutritional status (albumin, vitamin D, and BUN). Given that the association between malnutrition and hip fracture is established, future studies should deepen the analysis of nutritional parameters in order to plan an adequate population intervention in a systematic way. The data for this study were collected according to the National Hip Fracture Registry, which does not include the collection of nutritional data. We consider it essential to consider its future inclusion, which will allow us to establish preoperative optimization measures, beyond the aforementioned population-based interventions. Other limitations of this study include a relatively small sample size (*n* = 124) for a common pathology such as hip fracture and the collection of patients exclusively from a rural setting. In addition, the participants were older and probably sicker than typical Spanish patients with hip fracture. This factor means that their measures of prevalence of vitamin D deficiency, probable malnutrition, and probable sarcopenia may be higher than in the general hip fracture population. This trait limits the generalizability and external validity of our findings. This study’s retrospective nature may also introduce bias due to the reliance on pre-existing records. This possible bias might also affect the validity of the findings. Future prospective studies are needed to confirm the findings of the present study. Finally, the ROC analysis for BUN as a mortality predictor shows relatively weak specificity (54.9%). This relatively weak specificity makes the utility of BUN as a predictor questionable, and its validity should be limited just as a screening method, but further investigations must be conducted to confirm the results. Moreover, the development of alternative statistical models that yield stronger predictors is also needed.

A multivariate analysis controlling for other potential confounders (e.g., age, sex, comorbidities, and surgical techniques) is lacking in the present study, as the small sample size did not allow us to obtain statistically significant findings in the multivariate analysis. Consequently, our results suggest associations, but these relationships observed must still be elucidated when controlling for confounding variables.

## 5. Conclusions

The rural population suffering from hip fracture, evaluated in the present study, showed significant data of malnutrition and nutritional deficiencies, including hypoalbuminemia in 37.1% of the patients, deficiency, or insufficiency of vitamin D in 94.3% and sarcopenia, as assessed by elevated BUN values in 82.8% of the cases. Postoperative mortality was 8.1% and showed a significant association with lower vitamin D levels and elevated BUN values. A BUN cut-off point of 28.9 mg/dL showed a sensitivity of 80% for predicting postoperative mortality; however, the low specificity of this marker (54.9%) limits its application as a preoperative predictor of increased mortality risk and might be only used as a screening tool, but further confirmation of the findings is required.

Consequently, an adequate nutritional assessment of patients with hip fracture is vital, as it is one of the most significant risk factors to consider regarding potential complications. Given the increase in hip fractures and their possible complications, such as high mortality or loss of functionality, as well as their relationship with malnutrition, it is deemed pertinent to evaluate the nutritional status of patients undergoing hip fracture surgery and its relationship with other complications such as sarcopenia and increased mortality. Understanding the current nutritional status of patients undergoing hip fracture surgery will help to determine appropriate strategies to improve their health and reduce potential complications.

## Figures and Tables

**Table 1:** Association between type of fracture, nutritional parameters, and mortality.

	BUN	Vit D	Albumin	Mortality
Intracapsular non-displaced (25–20.2%)	26.9 ± 6.4	9.6 ± 6.6	3.69 ± 0.4	8%
Pertrochanteric (37–29.8%)	30.5 ± 13.4	12.8 ± 10.6	3.59 ± 0.4	8.1%
Subtrochanteric (55–44.3%)	32.0 ± 14.9	13.3 ± 9.7	3.53 ± 0.4	7.4%
Intracapsular displaced (7–5.6%)	33.3 ± 8.0	13.3 ± 11.1	3.66 ± 0.4	14.3%
*p* value	0.529	0.551	0.566	0.455

**Table 2:** Association between surgical procedure, nutritional parameters, hospital stay, and mortality.

	BUN	Vit D	Albumin	Hospital Stay	Mortality
Intramedullary nail (63–50.8%)	32.2 ± 13.8	13.0 ± 9.5	3.56 ± 0.4	10.5 ± 4.0	8.6%
Partial prosthesis (26–20.9%)	28.3 ± 12.4	11.5 ± 8.0	3.64 ± 0.4	11.6 ± 4.2	3.8%
Total prosthesis (35–28.2%)	30.7 ± 10.3	11.3 ± 11.7	3.61 ± 0.3	9.0 ± 2.4	6.3%
*p* value	0.427	0.430	0.214	0.139	0.124

## Data Availability

Data are unavailable due to privacy or ethical restrictions.
